# Possible Mechanism(s) of the Relaxant Effects of *Achillea wilhelmsii* on Guinea-Pig Tracheal Chains

**Published:** 2013

**Authors:** Mohammad Hossein Boskabady, Naeima Eftekhar, Mahsa Kaveh

**Affiliations:** *Applied Physiology Research Center and Dept. of Physiology, School of Medicine, Mashhad University of Medical Sciences, Mashhad, Iran. *

**Keywords:** *Achillea wilhelmsii*, Hydroalcholc extract, Possible mechanism, Relaxant effects, Guinea pig, Trachea

## Abstract

*Achillea wilhelmsii *have been used in folk remedies. The relaxant effects of the extract of *A. wilhelmsii *on tracheal chains of guinea pigs were examined. The relaxant effects of four cumulative concentrations of the extract, theophylline and saline were examined by their relaxant effects on precontracted tracheal chains of guinea pig by KCl (group 1), 10 μM methacholine (group 2), incubated tissues by atropine, propranolol and chlorpheniramine and contracted by KCl (group 3) and incubated tissues by propranolol and chlorpheniramine and contracted by methacholine (group 4). In group 1 and 2, all concentrations of theophylline and three higher concentrations (4, 6 and 8 mg/mL) of the extract showed significant relaxant effects compared to that of saline. In groups 3 and 4 experiments also all concentrations of the extract showed significant relaxant effects compared to that of saline. The relaxant effect of three higher concentrations (4, 6 and 8 mg/mL) of the extract in group 1 were significantly greater than those of group 2 and in group 3 were significantly lower than those of group 1. There were significant positive correlations between the relaxant effects and concentrations for theophylline in groups 1 and 2 and the extract in all four groups of experiments. These results showed a potent relaxant effect for the extract from *A. wilhelmsii *on tracheal chains of guinea pigs. A muscarinc receptor blockade was also suggested for the extract.

## Introduction

The herb *Achillea*, which belongs to the family Compositae (Asteraceae), is a genus with more than 100 species all around the world (1). These plants are medicinal perennial rhizomous herbs that are native to Europe and Western Asia, although they are also found in Australia, New Zealand and North America (2, 3). *Achillea millefolium C. Koch *is the best known species, commonly named yarrow, and was used as traditional medicine in Europe and by native Americans for treating swollen tissues and wounds (4-6). 

The aerial parts of different species of the genus are widely used in folk medicine due to numerous medicinal properties, such as anti-inflammatory, antispasmodic, antihemorrhoidal, stomachic and antiseptic (7–9). 

The main components of the oil of *A. wilhelmsii *are carvacrol (25.1%), linalool (11.0%), 1,8-cineol (10.3%), E-nerolidol (9.0%) and borneol (6.4%) (10). The main components of the monoterpene fraction of the oil are camphor, borneol, linalool, 1,8-cineole, chrysanthenol acetate and carvacrol (9).

The analgesic (11) and anticonvulsant (12) effects of the plant have been demonstrated. Several investigators (13, 14) reported significant effects from the extracts of *A. wilhelmsii *or *A. talagonica *grown in Iran as being antilipidemic, antihypertensive or immunosuppressive for humans or laboratory animals respectively. The relative radical scavenging activity of the plant is also documented (15). 

The antispasmodic activity of total extract of *A. nobilis *subsp. sipylea on rat duodenum was observed (16). Some flavonoids can act as spasmolytic agents by relaxing various smooth muscles (17, 18). They have also reduced the tone of guinea-pig isolated trachea, main pulmonary artery, rat uterus and rat vas deferens (19-21). It has been also suggested that the inhibitory effects of cirsiliol on smooth muscle are attributed to inhibition of transmembrane Ca^2+^ influx (22). In addition, the relaxant effect of carvacrol on tracheal smooth muscle was observed in our previous study (23).

Therefore in the present study, the possible mechanism(s) of relaxant effects of hydro alcholic extract from *A. wilhelmsii *on tracheal chains of guinea pigs were examined.

## Experimental

*Plant and extraction*

*Achillea wilhelmsii *was collected form north east region of Iran and identified by MR Joharghi. A voucher specimen was preserved in the Herbarium of the School of Agriculture, Ferdowsi University (Herbarium No: 40377, FUMH). The plant was dried in the absence of sun light. The hydro-alcoholic extract of the plant was prepared as follows: fifty grams of *Achillea millefollium *were grinded, added to 700 mL of ethanol 50% (350 mL of pure ethanol was diluted with water to final volume of 700 mL) using the Soxhlet apparatus. The solvent was then removed under reduced pressure. The plant ingredient concentration in the final extract was adjusted to 0.1 g/mL by adding distilled water to the dried extract. Three and half (3.5) grams of extract was collected from 50 grams of the grinded plant. 

*Tissue preparation*

Guinea pigs (400-700 g, both sexes) were killed by a blow on the neck and tracheas were removed. Each trachea was cut into 10 rings (each containing 2-3 cartilaginous rings). The study was approved by Ethical Committee of Mashhad University of Medical Sciences. Before experiments, animals were housed in individual cages with access to food and water ad libitum and were maintained at 22º ± 2ºC on a 12 h light/dark cycle (light period 0700 and 1900 h). All the rings were then cut open opposite the trachealis muscle, and sutured together to form a tracheal chain (24, 25). Tissue was then suspended in a 10 mL organ bath (organ bath 61300, Bio Science Palmer-Washington, Sheerness, Kent U.K.) containing Krebs-Henseliet solution of the following composition (mM): NaCl 120, NaHCO_3_ 25, MgSO_4_ 0.5, KH_2_PO_4_ 1.2, KCl 4.72, CaCl_2_ 2.5 and dextrose 11.

The Krebs solution was maintained at 37°C and gassed with 95% O_2_ and 5% CO_2_. Tissue was suspended under an isotonic tension of 1 g and allowed to equilibrate for at least 1 h while it was washed with Krebs solution every 15 min.

*Protocols*

The relaxant effects of four cumulative concentrations of the extract of *A. wilhelmsii *(2, 4, 6 and 8 mg/mL) and theophylline anhydrous (Sigma Chemical Ltd UK) (0.2, 0.4, 0.6 and 0.8 mM) as positive control, and saline (1 mL) as negative control were examined. To produce the first concentration of the extract, 0.2 mL of 10 g% was added to a 10 mL organ bath and for other three concentrations; 0.2 mL of 10 g% was added to organ bath three times respectively. For theophylline, 0.2 mL of 0.01 M concentrated solution was added to organ bath 4 times. The consecutive volumes were added to organ bath at five minutes intervals.

In each experiment, the effect of saline, four cumulative concentrations from the extract and theophylline on contracted tracheal smooth muscle was measured after exposing tissue to each concentration of the solution for 5 min. A decrease in tone was considered to be a relaxant (bronchodilatory) effect and expressed as positive percentage change in proportion to the maximum contraction. An increase in tone was considered as a contractile (bronchoconstrictory) effect which was expressed as negative percentage change (24, 25).

The relaxant effect of different solutions was tested with four different experimental designs, as follows:

I: On tracheal chains contracted by 60 mM KCl (group 1 experiment, n= 7).

II: On tracheal chains contracted by 10 μM methacholine hydrochloride (Sigma Chemical Ltd UK), (group 2 experiment, n= 9).

III: On tracheal chains incubated with 1 μM atropine, propranolol and chlorpheniramine and contracted by 60 mM KCl (group 3 experiment, n= 7).

IV: On tracheal chains incubated with 1 μM propranolol and chlorpheniramine and contracted by 10 μM methacholine (group 4 experiment, n= 8).

The relaxant effects in four groups of experiments were examined in four different series of tracheal chains. All of the experiments were performed randomly with a 1 h resting period of tracheal chains between each two experiments while washing the tissues every 15 min with Krebs solution. In all experiments contractions were measured using an isotonic transducer (Harvard APP LTD, 50-6360 SINO. 0210) and measured by using a software by a computer )Acer model NO.: G781) recording.

*Statistical analysis*

All data were expressed as mean±SEM. The data of relaxant effects obtained in four groups of experiments were compared using one way analysis of variance (ANOVA) and Tukey-Kramer post test. The relaxant effect of the extract and theophylline were related to the concentrations using least square regression. Significance was accepted at p < 0.05.

## Results

*Relaxant effect*

In group 1 (non-incubated tissues contracted by KCl) and 2 (non-incubated tissues contracted by methacholine), all concentrations of theophylline and three higher concentrations (4, 6 and 8 mg/mL) of the extract showed relaxant effects compared to that of saline (p < 0.001 for all cases) (Tables 1 and 2).

In groups 3 and 4 experiments also all concentrations of the extract showed relaxant effects compared to that of saline (p < 0.01 to p < 0.001; Table 3).

*Comparison of the relaxant effect of theophylline with that of the extract*

In groups 1, the relaxant effect of only lowest concentration (2 mg/mL) and in group 2 the relaxant effect of three lower concentrations (2, 4 and 6 mg/mL) of the extract were significantly less than those of theophylline (p < 0.05 to p < 0.001; Tables 1 and 2).

**Table 1:** Relaxant effect of the extract of *Achillea millefollium *(percentage change in proportion to the maximum contraction) in comparison with negative control (saline) and positive control (theophylline) in group 1 experiments (contracted tracheal chains with 60 mM KCl, n = 7).

**Different Concentration**	**Saline**	**Theo.**	**St. Dif. ** ***vs *** **Saline**	**Extract**	**St. Dif. ** ***vs *** **Saline**	**St. Dif. ** ***vs *** **Theo.**
1	-	28.25 ± 3.24	p < 0.001	0.24 ± 7.29	NS	p < 0.001
2	-	49.04 ± 4.19	p < 0.001	42.32 ± 6.84	p < 0.001	NS
3	-	75.62 ± 5.31	p < 0.001	80.3 ± 11.13	p < 0.001	NS
4	0.43 ± 0.2	88.33 ± 8.16	p < 0.001	108.71 ± 13.71	p < 0.001	NS

Values are presented as mean±SEM. St. Dif.: Statistical deference. The four different concentrations for the extract were 2, 4, 6 and 8 mg/mL, and for theophylline, 0.2, 0.4., 0.6 and 0.8 mM. Theo.: theophylline

**Table 2:** Relaxant effect of the extract of *Achillea millefollium *(percentage change in proportion to the maximum contraction) in comparison with negative control (saline) and positive control (theophylline) in group 2 experiments (contracted tracheal chains by 10 μM methacholie, n = 9)

**Different Concentration**	**Saline**	**Theo.**	**St. Dif. ** ***vs *** **Saline**	**Extract**	**St. Dif. ** ***vs *** **Saline**	**St. Dif. ** ***vs *** **Theo.**
1	-	30.33 ± 3.26	p < 0.001	0.58 ± 3.28	NS	p < 0.001
2	-	48.24 ± 4.93	p < 0.001	22.6 ± 2.55	p < 0.001	p < 0.001
3	-	68.02 ± 5.08	p < 0.001	49.27 ± 5.94	p < 0.001	p < 0.05
4	0.6 ± 0.23	90.86 ± 6.01	p < 0.001	87.98 ± 8.60	p < 0.001	NS

For abbreviations see Table 1

*Comparison of the relaxant effect between group 1 and 2*

**Figure 1:**
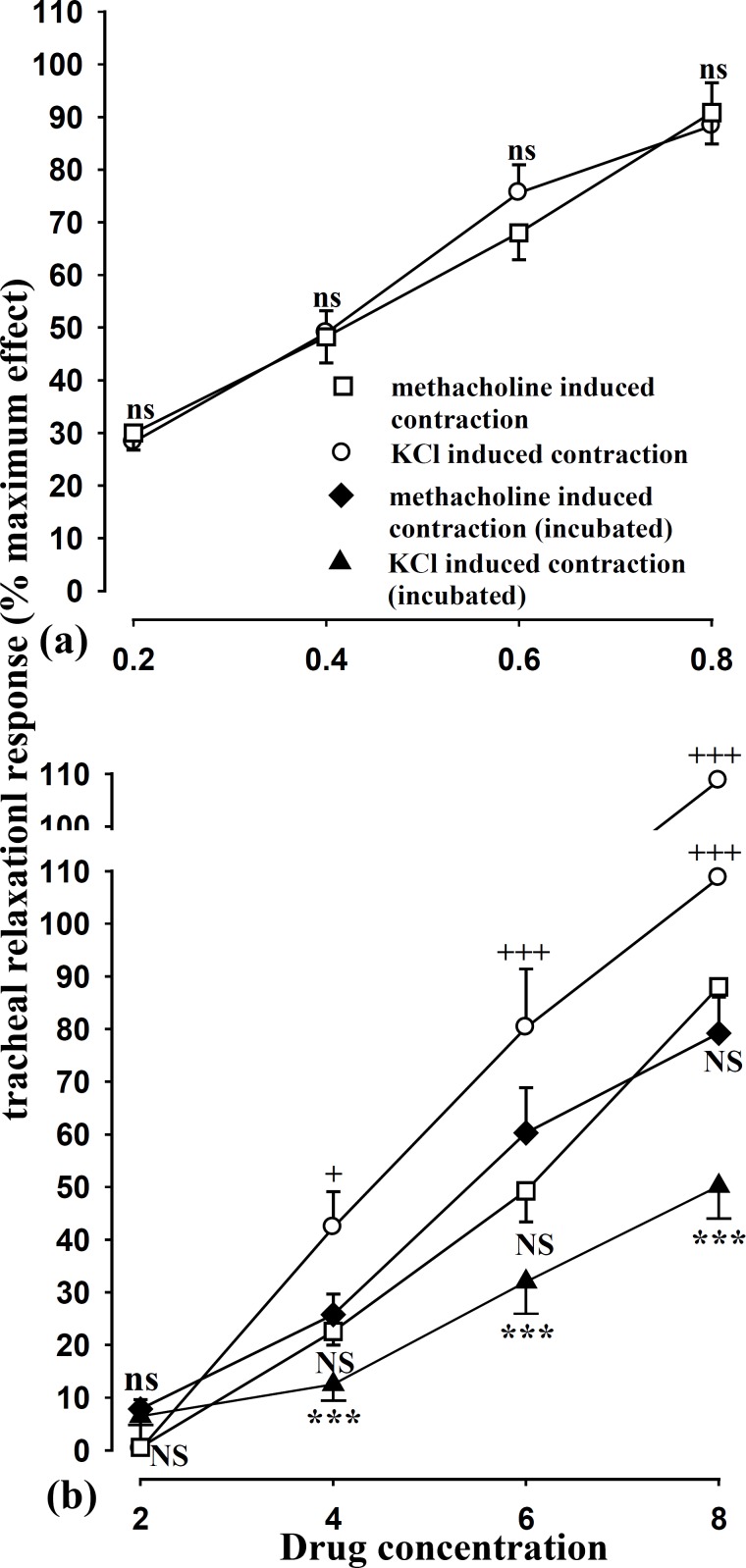
Concentration response curves of the relaxant effect of theophylline (a) and the extract of *Achillea millefollium *(b) in four groups of experiments. group 1; KCl induced contraction of tracheal chains (ο, n = 7), group 2; methacholine induced contraction of tracheal chains (■, n = 9), group 3; KCl induced contraction of incubated tracheal chains with atropine, propranolol and chlorpheniramine (▲, n = 7) and group 4; methacholine induced contraction of incubated tracheal chains with propranolol and chlorpheniramine (♦, n = 8). Statistical differences in the relaxant effect of different concentrations of each solution between group 1 and 2; ns: non-significant difference, +; p < 0.05, +++; p<0.001. Statistical differences in the relaxant effect of different concentrations of the extract between group 1 and 3; ns, non significant difference, ***; p < 0.001. Statistical differences in the relaxant effect of different concentrations of the extract between group 2 with those of group 4; NS, non significant difference. The concentration unit for the extract was mg/mL and for theophylline, mM.

The relaxant effect of three higher concentrations (4, 6 and 8 mg/mL) of the extract in group 1 (contracted tracheal muscle with KCl) were greater than those of group 2 (contracted tracheal muscle with methacholine), (p < 0.01 to p < 0.001). However, the relaxant effect of theophylline between two groups was not statistically sugnificant (Figure 1).

*Comparison of the relaxant effect between incubated and non-incubated tissues*

The relaxant effect of three higher concentrations (4, 6 and 8 mg/mL) of the extract in group 3 (tracheal muscle incubated with atropine, propranolol and chlorpheniramine and contracted with KCl) were significantly lower than those of non incubated tissues contracted by KCl (p < 0.05 to p < 0.001). However there was no significant difference in relaxant effect between non incubated (group 2) and incubated (incubated tissues with propranolol and chlorpheniramine, group 4) tissues contracted by methacholine (Figure 2b).

*Comparison of the relaxant effect between incubated tissues contracted by KCl and methacholine*

The relaxant effect of three higher concentrations (4, 6 and 8 mg/mL) of the extract in group 3 (tracheal muscle incubated with atropine, propranolol and chlorpheniramine) contracted with KCl were lower than group 4 (incubated tissues with propranolol and chlorpheniramine) contracted by methacholine; (p < 0.05 to p < 0.01; Table 3).

**Table 3:** Relaxant effect of the extract of *Achillea millefollium *(percentage change in proportion to the maximum contraction) in comparison with negative control (saline) and positive control (theophylline) in group 3 experiments (contracted tracheal chains by 60 mM KCl-Inc, n = 7) and group 4 experiments (contracted tracheal chains by 10 μM methacholie-Inc, n = 8)

**Different Concentration**	**Saline**	**KCl-Inc**	**St. Dif. ** ***vs *** **Saline**	**M-Inc**	**St. Dif. ** ***vs *** **Saline**	**St. Dif. KCl-Inc ** ***vs *** **M-Inc**
1	-	6.47 ± 1.64	p < 0.01	7.84 ± 1.81	p < 0.01	NS
2	-	12.54 ± 3.12	p < 0.01	25.75 ± 3.92	p < 0.001	p < 0.05
3	-	32.00 ± 6.04	p < 0.005	60.27 ± 8.62	p < 0.001	p < 0.05
4	0.72 ± 0.18	50.17 ± 6.11	p < 0.001	79.18 ± 6.91	p < 0.001	p < 0.01

For abbreviations see Table 1

*Correlation between concentrations of solutions and their relaxant effect*

There were significant positive correlations between the relaxant effects and concentrations for theophylline in groups 1 and 2 and the extract in all four groups of experiments (p < 0.001 for all cases; Table 4).

**Table 4:** Correlation (r) between the relaxant effect s of the extract of *Achillea millefollium *and theophylline with their concentrations in four groups of experiments.

**Different Solutions**	**KCl**		**Metha**		**KCl-Inc**		**Meth-Inc**	
	**R**	**p-value**	**R**	**p-value**	**R**	**p-value**	**r**	**p--value**
Extract	0.8507	p< 0.001	0.891	p< 0.001	0.664	P = 0.001	0.735	p< 0.001
Theo.	0.8396	p< 0.001	0.850	p< 0.001				

## Discussion

In this study, the relaxant effect of the extract from *A. wilhelmsii *in comparison with saline as negative control and theophylline as positive control was studied. In group 1 (contracted tracheal chains with KCl), the extract and theophylline showed relaxant effect on tracheal smooth muscle. The relaxant effect of only lower concentration of the extract was significantly less than that of theophylline.

The results of this study confirmed those of the study of Asgari *et al*. (14) indicating the antihypertensive effect of the plant which is perhaps due to its relaxant effect on vascular smooth muscle.

In group 2 (contracted tracheal chains with methacholine), the extract and theophylline showed relaxant effect on tracheal smooth muscle. The relaxant effects of three lower concentrations of the extract were significantly less than that of theophylline. In addition, the relaxant effects of three higher concentrations of the extract in this group were significantly lower than those of group 1.

The greater relaxant effect of the extract on KCl induced contraction of tracheal muscle (group 1), indicates that the extract may have inhibitory effect on calcium channels and/or opening effect on potassium channels. In fact, the relaxant effect of potassium channels openers (26) and calcium channel blockers (27) has been shown. In addition, it is well known that KCl can affect calcium channels. However, the greater relaxant effect on KCl induced contraction (group 1) compared to methacholine induced contraction (group 2) is in favour of inhibitory effect of the extract on calcium channels. In fact, these results supported by those of previous studies suggesting that the inhibitory effects of cirsiliol, a flavone isolated from *Achillea fragrantissima*, on smooth muscle are attributed to inhibition of transmembrane Ca^2+^ influx (22).

It is known that the agonists of *β*-adrenergic receptors are the most potent relaxant agents of tracheal smooth muscle (28). However, no difference in the relaxant effect of the extract between non incubated (group 2) and incubated tracheal smooth muscle with propranolol and chlorpheniramine (group 4) and contracted with methacoline indicates that the extract has no stimulatory effect on *β*-adrenergic receptors. These results also suggest that the relaxant effect is not mediated by the inhibitory effect of the extract on histamine (H1) receptors*.*

However, the significant lower relaxant effect on incubated tracheal smooth musele with propranolol, chlorpheniramine and atropine and contracted with KCl (group 3) compared to non incubated tissues (group 1) indicated a muscarinic inhibitory effect for the extract. In addition, the relaxant effects of muscarinic receptors inhibitory agents have been documented previously (29). However, more studies are required to examine the exact mechanism(s) of the relaxant effect seen for the extract of the plant.

The extract contained several substances, which could be lead to relaxation response by several mechanisms. Therefore, in group 3, tracheal smooth muscle were incubated by atropine, chlorpheniramine and propranolol to inhibit muscarinic, histamine (H1) and adrenergic receptors and evaluate the possible contribution of these three sets of receptors in observed relaxation response. In this group a significant reduction in relaxation response was observed. In group 4, tracheal smooth muscle was incubated with chlorpheniramine and propranolol to inhibit histamine (H1) and adrenergic receptors and leave muscrinic receptor intact. In this group the relaxation response did not changed significantly which suggest that main mechanism of the relaxant effect of the extract is its blocking effect on muscarinic receptors. However, the exact mechanism(s) of the relaxant effect of the plant should be investigated in further studies.

One of the constituent of *Achillea wilhelmsii *is carvacrol which its relaxant effect was shown previously (23, 30). Therefore, the relaxant effect of the plant could be due to its main constituent, carvacrol. However, the relaxant effect of carvacrol was higher on methacholine induced contraction of tracheal smooth muscle compared to KCl induced contraction but the effect of the extract of *Achillea wilhelmsii *was more potent on KCl induced contraction. These results indicate that the mechanism(s) of the relaxant effect of the extract is not exactly the same as that of carvacrol. Therefore, the relaxant of the extract of *Achillea wilhelmsii *is not just due to its constituent carvacrol.

There were positive correlations between concentrations and the relaxant effects of both theophylline and the extract indicating a concentration dependent relaxant effect for the extract as well as for theophylline. Although the relaxation effect of the extract of other species of this plants family was shown in different types of smooth muscle (19-21), the relaxant effect of *A. wilhelmsii *on tracheal smooth muscle which is in some way different from other smooth muscle had been studied and this is the first report in this regard. The relaxant effects of the extract observed in the present study can be translated to bronchodilation and might have potential benefits in asthmatic patients.

In conclusion the results of this study indicated a potent relaxant effect for the extract from *A. wilhelmsii *on tracheal chains of guinea pigs. The results also suggest that the H1 histamine receptors and Beta-adrenergic receptors are not involved in the relaxant effect of the extract.
